# Supplementary material for: Musculoskeletal modelling of an ostrich (Struthio camelus) pelvic limb: influence of limb orientation on muscular capacity during locomotion
Source: PeerJ. 2015 Jun 11;3:e1001. doi: 10.7717/peerj.1001 (PMC4465956; doi:10.7717/peerj.1001)
Supplement: Text S1 — Simplifications of pelvic limb muscle anatomy in the musculoskeletal model. [file peerj-03-1001-s001.docx]

**Hutchinson et al. “Musculoskeletal modelling of an ostrich (*Struthio camelus*) pelvic limb: Influence of limb orientation on muscular capacity during locomotion”**

**PeerJ manuscript, Supplementary Text**

Simplifications of Pelvic Limb Muscle Anatomy in the Musculoskeletal Model

Some muscles were combined as compound units in the model, including the heads of: M. iliotibialis cranialis (IC), Mm. obturatorii (OM), Mm. puboischiofemorales (PIFML), Mm. caudofemorales (CFP; pars caudalis’s added to pelvica’s two main parts), Mm. femorotibiales (proximal and distal [also pectineal, accessorius; Zinoviev, 2006] heads combined to form three muscles: FMTL, FMTIM, FMTM), M. gastrocnemius partes medialis (GM; included “supramedialis” which is largely tendinous), M. extensor digitorum longus (EDL), and M. fibularis longus (FL; given tendon extending to the digits but this would only be taut at some joint positions; see Gangl et al., 2004 and Schaller et al., 2009). The origins of the different digital flexor groups (FHL, FPPD3, FPD3, FPD4) were combined into single origins of intermediate positions, as they were too complex to model individually. As Table 4 notes, this means that some muscles did not have actions about the knee that some of their parts would have.

As noted in the Methods (also shown in Table 2), three larger muscles were split into two heads: M. iliotibialis lateralis (ILa,ILp), M. iliofibularis (ILFBa,ILFBp), M. iliotrochantericus caudalis (ITCa,ITCp). There is additional justification for this from anatomical studies of ostriches—e.g. two crura of M. iliotibialis lateralis and M. iliofibularis (Gangl et al., 2004)– and from biomechanical studies of birds—e.g. different length changes of cranial and caudal heads of M. iliotibialis lateralis (Carr et al., 2011).

Muscles that were omitted included M. fibularis brevis (tendinous), M. plantaris (tendinous), and M. popliteus (crosses fibula and tibia; not given a joint in our model, but see Fuss, 1996). Additionally, as in most other biomechanical studies of ostriches, we did not consider the small intrinsic muscles of the pes (Mm. extensores digiti, M. abductor digit IV, M. lumbricalis), whose contributions to toe flexor/extensor moments are presumably small compared with the long flexors/extensors (e.g., M. flexor digitorum longus; M. extensor digitorum longus). Schaller et al. (20011) present some investigations of these muscles that could be added to our model if internal functions of the feet were intended to be investigated and sufficient kinematic/anatomical data were collected. Haughton (1864) inferred that M. ambiens (AMB2 in our model; his “rectus femoris”) linked the hips and toes via tendinous connections to the digital flexors, providing the capacity to rapidly flex the limb in late stance as “one of the most striking phenomena in nature” according to Haugton (1864:p. 61). We did not include connections of this muscle to the toes because our dissections suggest these to be somewhat flimsy as well as difficult to characterize in a model, but this question may deserve re-examination with experimental analyses that test the tissues extending distally from the AMB2 muscle.

Our goal was not to include comprehensive anatomical detail in the model such as the complex interrelationships of some lower leg tendons – this was impossible. Rather, we aimed to capture the basic biomechanical aspects of pelvic limb muscle anatomy in ostriches, especially the major muscles responsible for limb support and movement, as a starting point enabling more detailed future analyses.
