# Supplementary figures and images for: Musculoskeletal modelling of an ostrich (Struthio camelus) pelvic limb: influence of limb orientation on muscular capacity during locomotion
Source: PeerJ. 2015 Jun 11;3:e1001. doi: 10.7717/peerj.1001 (PMC4465956; doi:10.7717/peerj.1001)

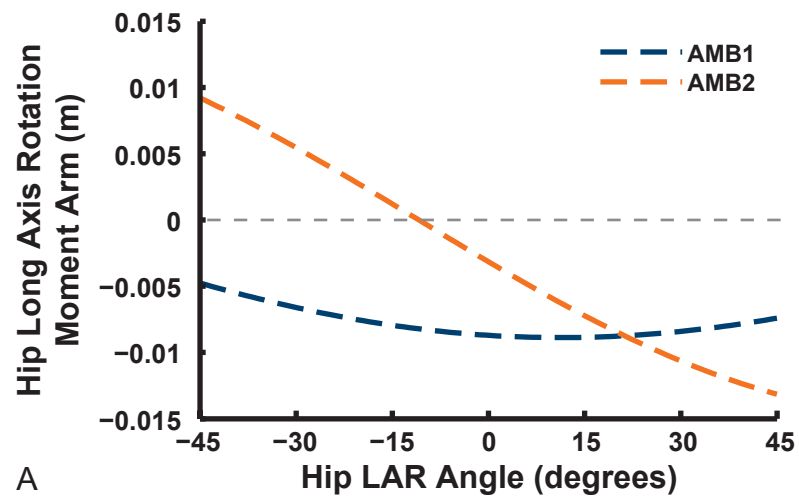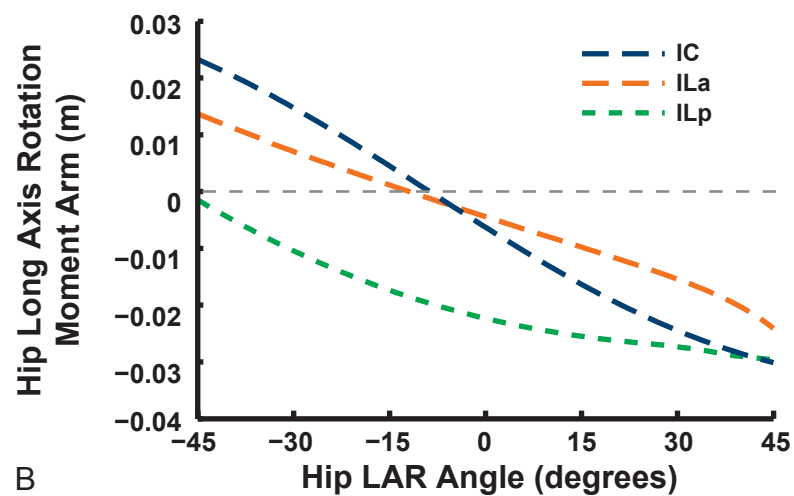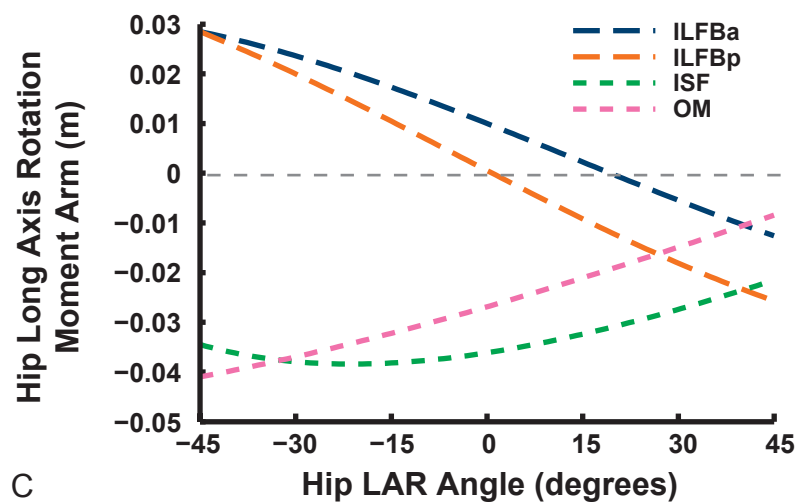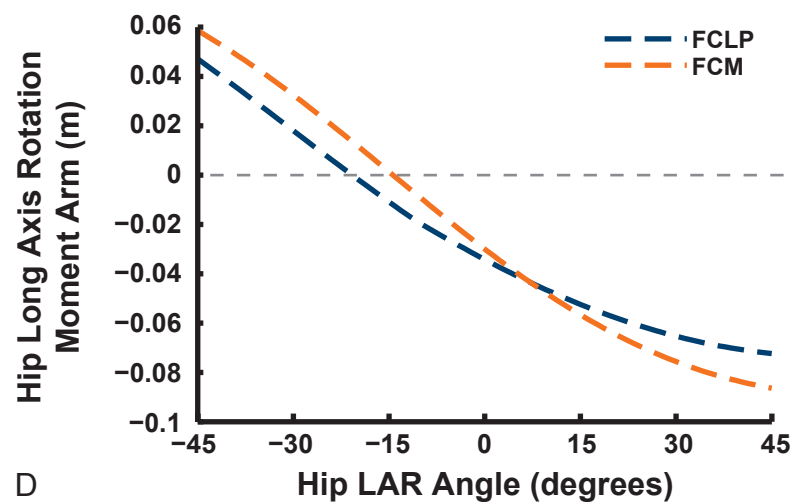

Supplement: Figure S1 — Hip muscle moment arms in long-axis rotation (LAR) or ab/adduction plotted against hip LAR or ab/adduction angles (cf. Figs. 12–15 plotted against hip flexion/extension angles), for key proximal thigh muscles. See caption for Fig. 9. [file peerj-03-1001-s002.pdf]

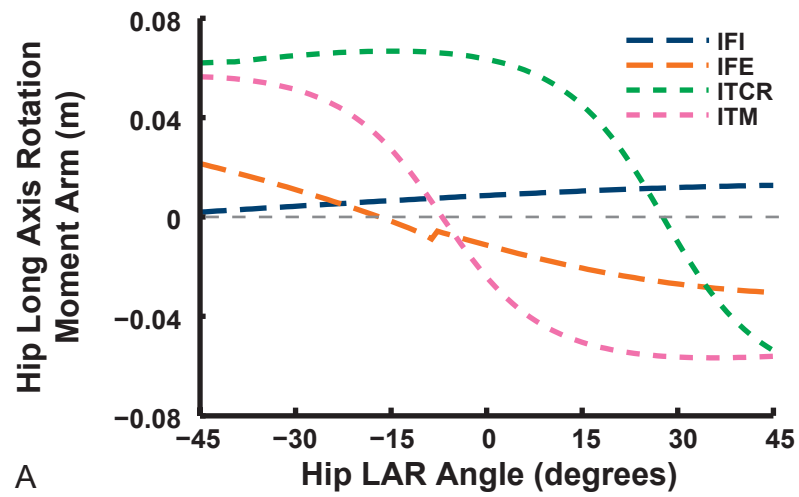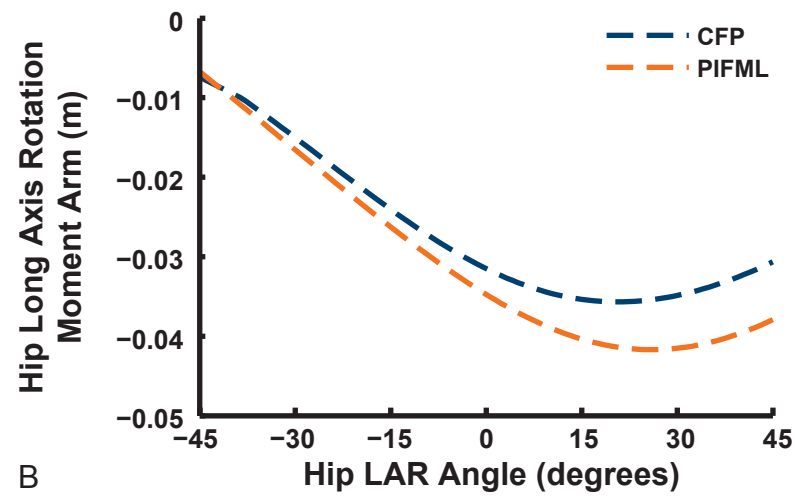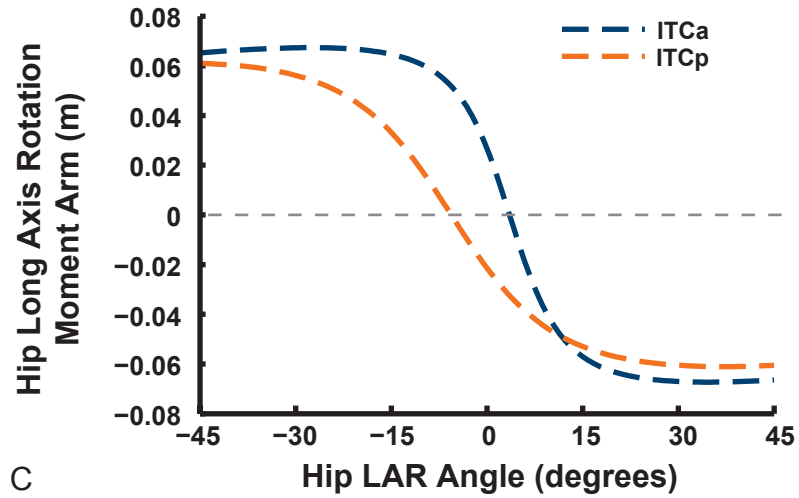

Supplement: Figure S2 — Hip muscle moment arms in long-axis rotation (LAR) or ab/adduction plotted against hip LAR or ab/adduction angles (cf. Figs. 12–15 plotted against hip flexion/extension angles), for key proximal thigh muscles. See caption for Fig. 9. [file peerj-03-1001-s003.pdf]

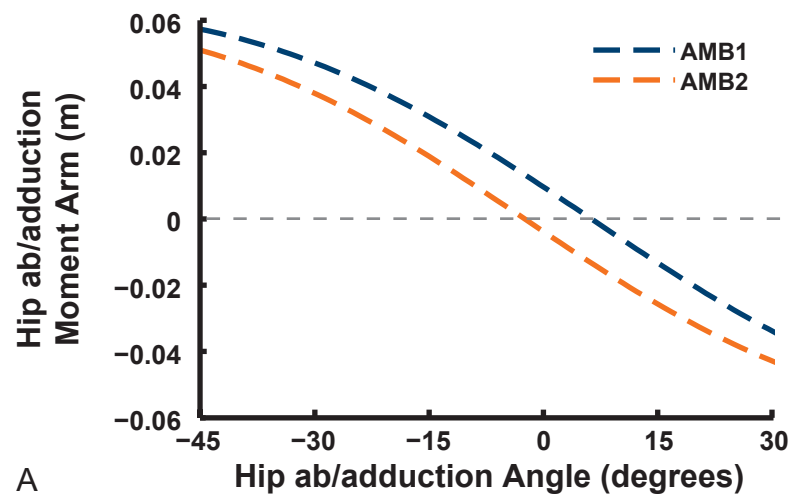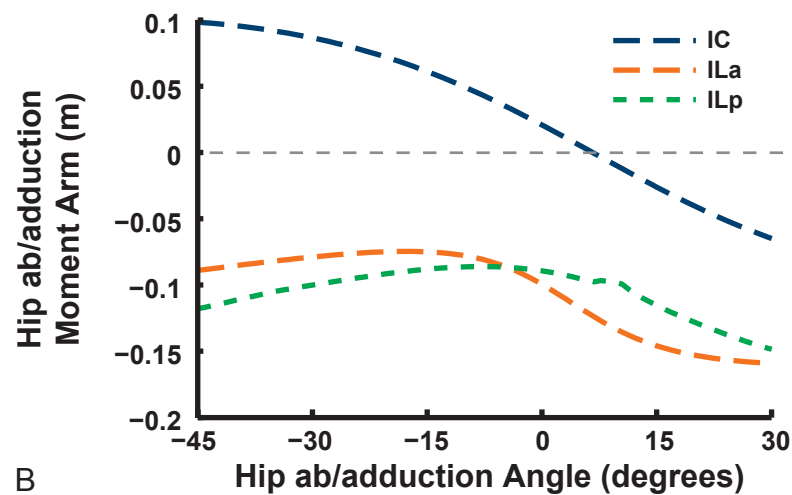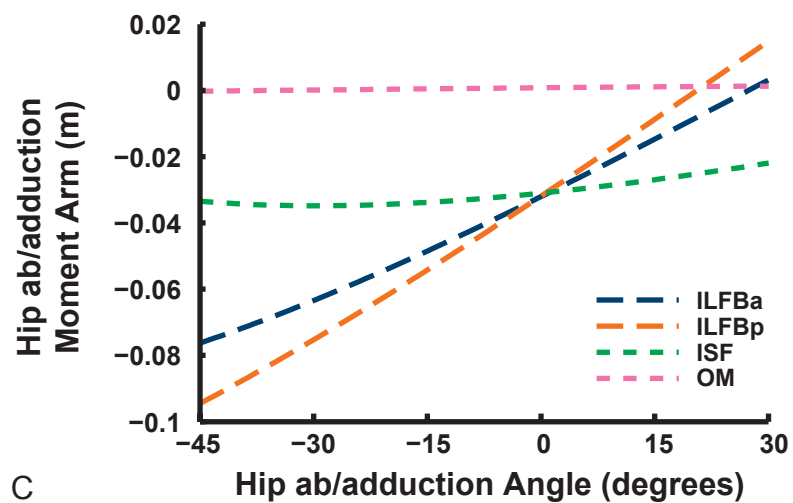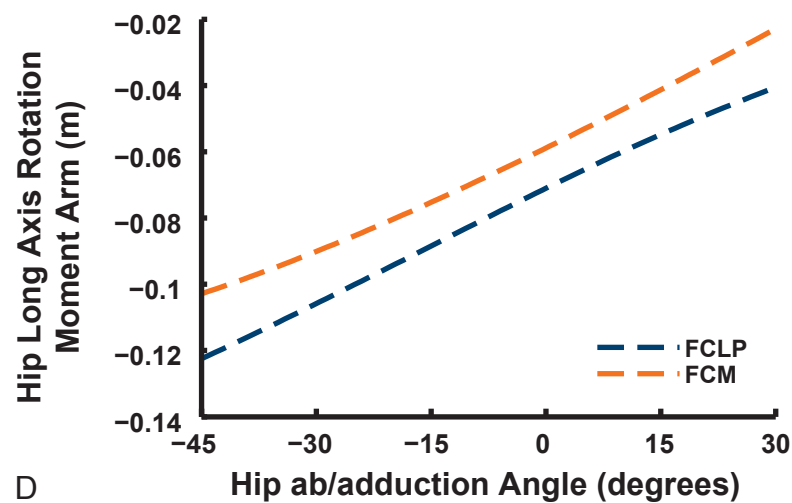

Supplement: Figure S3 — Hip muscle moment arms in long-axis rotation (LAR) or ab/adduction plotted against hip LAR or ab/adduction angles (cf. Figs. 12–15 plotted against hip flexion/extension angles), for key proximal thigh muscles. See caption for Fig. 9. [file peerj-03-1001-s004.pdf]

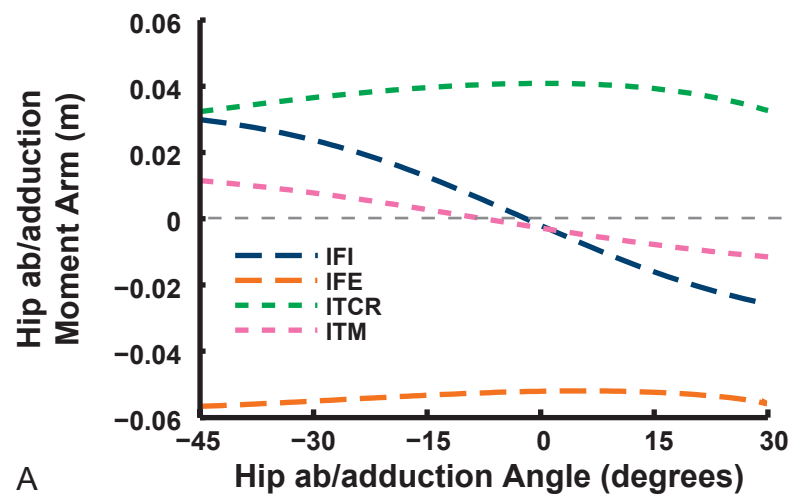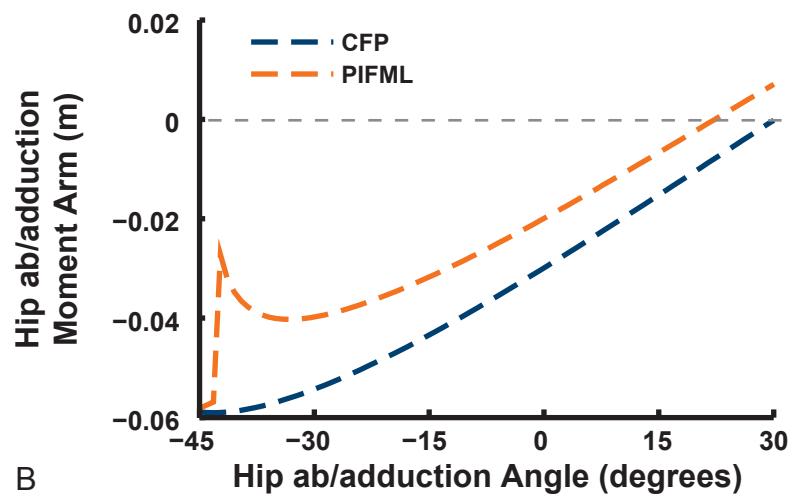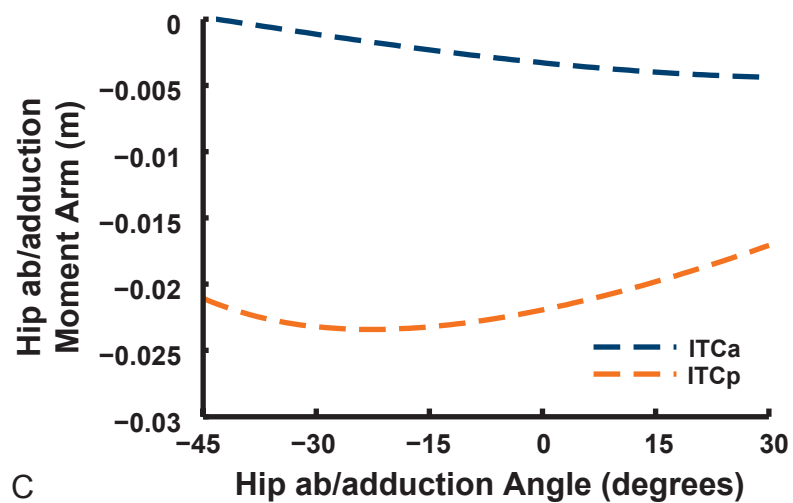

Supplement: Figure S4 — Hip muscle moment arms in long-axis rotation (LAR) or ab/adduction plotted against hip LAR or ab/adduction angles (cf. Figs. 12–15 plotted against hip flexion/extension angles), for key proximal thigh muscles. See caption for Fig. 9. [file peerj-03-1001-s005.pdf]

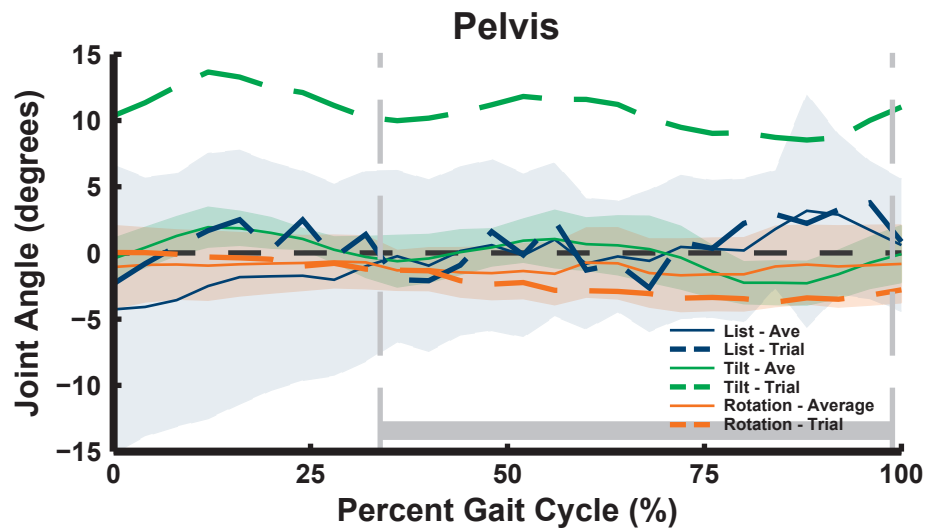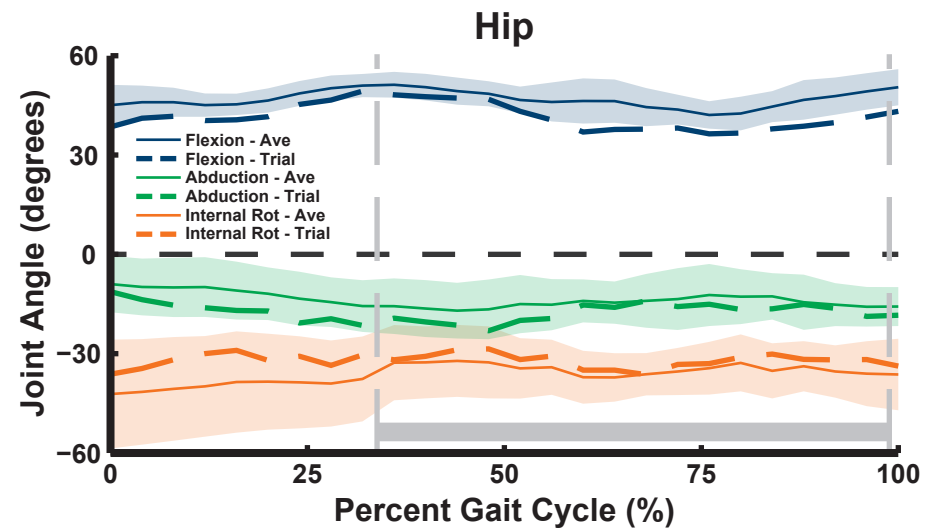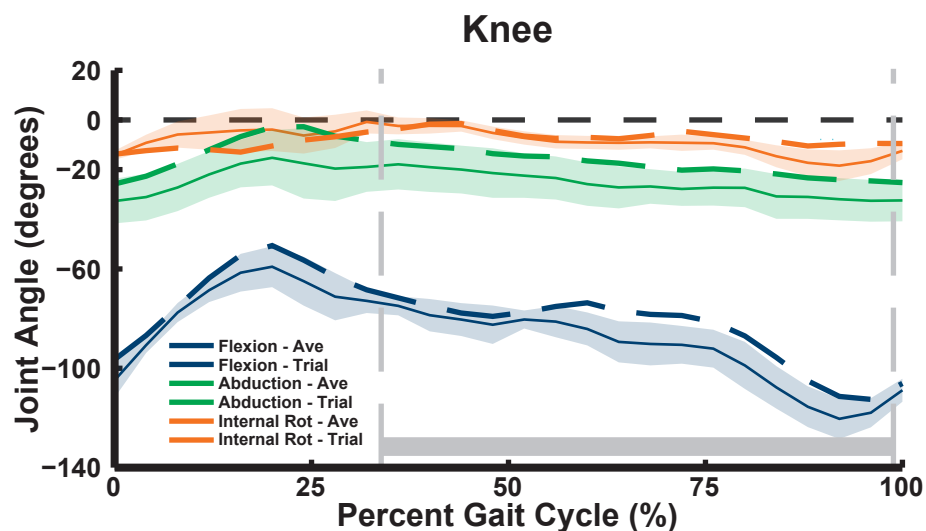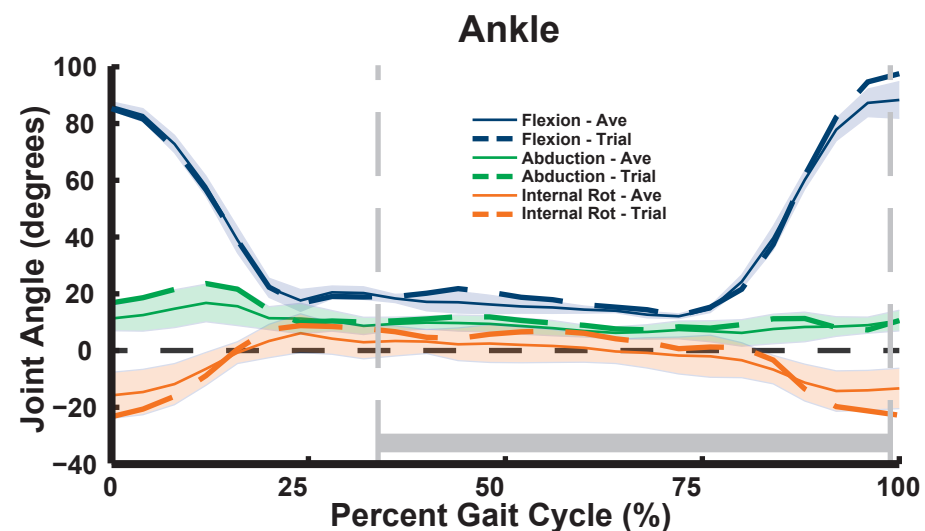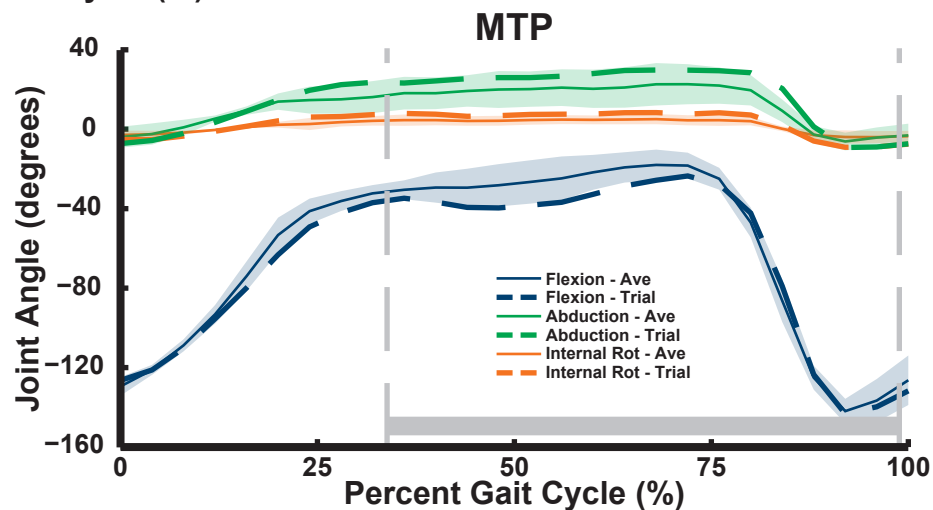

Supplement: Figure S5 — Kinematic data for walking ostriches, from the experiments by Rubenson et al. (2007), which solely reported the running data but also obtained these walking data. Joint angles for the pelvis, hip, knee, ankle and metatarsophalangeal (MTP) joint are reported, with shaded bars representing ±1 SD from the means (lines- “Ave” notation”). Thicker dashed lines representing the single walking trial used as a representative trial here. These representative trial data were used in Figs. 6–8. Vertical dashed lines represent foot-ground contact (left side) and foot liftoff (right side; at 100% of the gait cycle), with shaded horizontal bars indicating the stance phase. Joint angle conventions and terms follow Rubenson et al. (2007)- note that “internal rot” here corresponds to medial rotation in this study. [file peerj-03-1001-s006.pdf]
